# Lamellar corneal injury by bamboo splinters: a case report

**DOI:** 10.1186/1752-1947-3-7226

**Published:** 2009-05-08

**Authors:** Motoko Kawashima, Tetsuya Kawakita, Chika Shigeyasu, Jun Shimazaki

**Affiliations:** 1 Department of Ophthalmology, Tokyo Dental College, Sugano 5-11-13, Ichikawa City, Chiba, Japan; 2 Department of Ophthalmology, Keio University School of Medicine, 35 Shinanomachi, Shinjukuku, Tokyo, Japan

## Abstract

**Introduction:**

We report an unusual case of corneal lamellar injury caused by long bamboo splinters.

**Case presentation:**

A 70-year-old Japanese man visited our hospital with a bamboo injury. Slit lamp examination revealed that a bundle of bamboo splinters had deeply penetrated the corneal stroma of the right eye from the nasal limbus. The splinters were approximately 8 mm in length, but had not perforated the anterior chamber. They were completely removed by superficial corneal incision alongside each splinter with no consequences. The eye has remained healed for 3 months postoperatively.

**Conclusion:**

The bamboo splinters did not perforate the anterior chamber, although they were long and hard enough to do so. This may be because the spatula-like shape and flexibility of the bamboo splinters allowed them to penetrate the lamellar layer of the corneal stroma with ease, but with no perforation of deeper tissue.

## Introduction

Bamboo has been reported as an unusual palpebral or orbital foreign body in ophthalmological studies [1,2]. Bamboo prefers a warm climate with high humidity, and is common throughout East Asia. These characteristics make it a breeding ground for a wide variety of microorganisms such as fungi and bacteria [3,4]. Therefore, there is a high concomitant risk of the development of vision-threatening infections with penetrating bamboo injury to ocular tissues. Here, we report a patient with ocular bamboo injury where its successful treatment may be explained by a bamboo-specific traumatic mechanism.

## Case presentation

**Figure 1:**
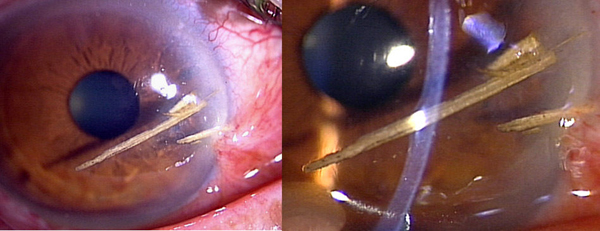
**Clinical findings at initial visit**. Bamboo splinters (approximate length 8 mm, width 1.5 mm) were lodged deeply in the stromal layer from the nasal side **(A)**. They had not, however, penetrated the stroma, and the anterior chamber was quiet **(B)**. The patient showed nasal corneal scars caused by previous pterygium resection in this eye. Vd = 0.08 (0.3× + 1.25 = cyl - 0.50A165).

A 70-year-old Japanese man visited our hospital in January 2007 with a foreign body lodged deeply in his ocular stroma. He had sustained this injury while felling bamboo. At the initial examination, his best-corrected visual acuity (BCVA) was 0.3 in the right eye and manifest refraction was +1.25D-0.50 × 165. Bamboo splinters, approximately 8 mm in length, had pierced the cornea from the nasal side (Figure 1), deeply penetrating the stroma. High magnification slit lamp examination revealed that, although they had deeply penetrated the stromal layer, they had not perforated the anterior chamber (Figure 1). The anterior chamber was of normal depth, with no aqueous leakage, and intraocular pressure was 12 mmHg.

To remove the splinters, an inclined corneal incision was created alongside each one. This incision allowed them to be removed completely and with ease, demonstrating an effective way of avoiding infection from residual splinters. The wound was sutured in place with four interrupted 10-0 nylon sutures after washing the interface. Corneal sutures were used to facilitate wound healing, and were removed completely after 1 week.

**Figure 2:**
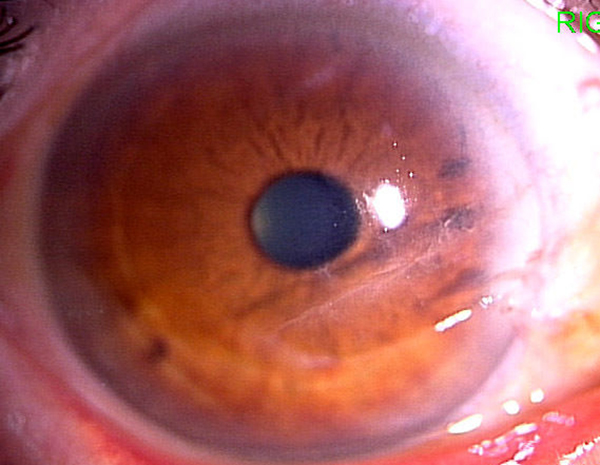
**Clinical findings 2 weeks postoperatively**. Although corneal sutures were performed to facilitate wound healing, they were removed completely after 1 week. The patient's eye showed no signs of infection and little scar formation; no strong astigmatism was observed. Vd = 0.4 (0.6× + 2.00 = cyl - 0.75A150).

The eye showed no signs of infection and little scar formation; no strong astigmatism was observed (Figure 2). BCVA was 0.6 and manifest refraction was +2.00D - 0.75 × 150. His eye has remained healed for 3 months postoperatively.

## Discussion

Bamboo injury is unusual, and few cases have been reported where penetration has occurred with no perforation of blood vessels in the neck [5] or perforation of the eye ball [1,2]. The combination of ample rigidity to allow penetration with sufficient flexibility to allow avoidance of perforation may offer a potential explanation for this phenomenon.

In our patient, the bamboo splinters were long and hard enough to penetrate the corneal stroma. It appears, though, that the physical properties specific to bamboo allow it to penetrate deeply, but with no perforation of the anterior chamber. We believe this phenomenon may also be partially explained by the spatula-like shape of bamboo, together with its flexibility, enabling it to break the lamellar layer of collagen fibers with ease.

The foreign body was lodged deeply in his stroma from the nasal limbus. We believe that the foreign body changed direction after coming into contact with the nose.

Morakotkarn *et al.* noted that bamboo acts as a huge reservoir of microorganisms, with 257 fungal strains being isolated from bamboo tissues in Japan [3]. Therefore, residual fine bamboo fragments may cause severe infection and haze [4]. This necessitates complete removal of such bamboo fibers to prevent infection, and especially fungal infection. In our patient, we were able to remove the bamboo splinters localized in the corneal stroma easily and completely by making an open inclined wound. This may be one of the reasons why the clinical course of this treatment was successful.

## Conclusion

The bamboo splinters were long and hard enough to penetrate the corneal stroma, but did not. This may be explained by the spatula-like shape and flexibility of the bamboo splinters enabling them to penetrate the lamellar phase with ease.

## Consent

Written informed consent was obtained from the patient for publication of this case report and any accompanying images. A copy of the written consent is available for review by the Editor-in-Chief of this journal.

## Competing interests

The authors declare that they have no competing interests.

## Authors Contributions

MK: study concept and design, patient care, drafting the manuscript, literature review, TK: editing the manuscript, CS: patient care, data collection, literature review, JS: study concept and design, revising the manuscript,

All authors have read and approved the final version of the manuscript.
